# Identification of Key Biomarkers and Candidate Molecules in Non-Small-Cell Lung Cancer by Integrated Bioinformatics Analysis

**DOI:** 10.1155/2023/6782732

**Published:** 2023-01-03

**Authors:** Liyan Yu, Xuemei Liang, Jianwei Wang, Guangxiang Ding, Jinhai Tang, Juan Xue, Xin He, Jingxuan Ge, Xianzhang Jin, Zhiyi Yang, Xianwei Li, Hehuan Yao, Hongtao Yin, Wu Liu, Shengchen Yin, Bing Sun, Junxiu Sheng

**Affiliations:** ^1^Department of Respiratory, The First Affiliated Hospital of Dalian Medical University, Dalian 116044, Liaoning Province, China; ^2^Department of Thoracic Surgery, First Affiliated Hospital, Dalian Medical University, Dalian 116044, China; ^3^Department of Radiation Oncology, First Affiliated Hospital, Dalian Medical University, Dalian 116044, China

## Abstract

**Background:**

Non-small cell lung cancer (NSCLC) is the most prevalent malignant tumor of the lung cancer, for which the molecular mechanisms remain unknown. In this study, we identified novel biomarkers associated with the pathogenesis of NSCLC aiming to provide new diagnostic and therapeutic approaches for NSCLC by bioinformatics analysis.

**Methods:**

From the Gene Expression Omnibus database, GSE118370 and GSE10072 microarray datasets were obtained. Identifying the differentially expressed genes (DEGs) between lung adenocarcinoma and normal samples was done. By using bioinformatics tools, a protein-protein interaction (PPI) network was constructed, modules were analyzed, and enrichment analyses were performed. The expression and prognostic values of 14 hub genes were validated by the GEPIA database, and the correlation between hub genes and survival in lung adenocarcinoma was assessed by UALCAN, *cBioPortal*, String and Cytoscape, and Timer tools.

**Results:**

We found three genes (PIK3R1, SPP1, and PECAM1) that have a clear correlation with OS in the lung adenocarcinoma patient. It has been found that lung adenocarcinoma exhibits high expression of SPP1 and that this has been associated with poor prognosis, while low expression of PECAM1 and PIK3R1 is associated with poor prognosis (*P* < 0.05). We also found that the expression of SPP1 was associated with miR-146a-5p, while the high expression of miR-146a-5p was related to good prognosis (*P* < 0.05). On the contrary, the lower miR-21-5p on upstream of PIK3R1 is associated with a higher surviving rate in cancer patients (*P* < 0.05). Finally, we found that the immune checkpoint genes CD274(PD-L1) and PDCD1LG2(PD-1) were also related to SPP1 in lung adenocarcinoma.

**Conclusions:**

The results indicated that SPP1 is a cancer promoter (oncogene), while PECAM1 and PIK3R1 are cancer suppressor genes. These genes take part in the regulation of biological activities in lung adenocarcinoma, which provides a basis for improving detection and immunotherapeutic targets for lung adenocarcinoma.

## 1. Introduction

There are 234,030 newly diagnosed lung cancer patients in 2018. Lung cancer is the leading cause of morbidity and mortality among malignant tumors, and the incidence and mortality rate are increasing year by year [1]. There are approximately 85% of lung cancer cases that are non-small cell lung cancer (NSCLC) [2]. It is still difficult to predict the prognosis for lung cancer despite the progress made in targeted drugs and immunotherapy. It is reported that the 5-year survival rate of NSCLC patients is less than 20%. The main treatments for NSCLC are surgery, chemotherapy, radiotherapy, targeted therapy, and immunotherapy. Chemotherapy and targeted drug therapy are the most common and effective treatments for NSCLC, especially for patients with recurrence and metastasis [3–5]. However, chemical antitumor drugs have great side effects, and patients are poorly tolerated. Targeted drugs combined with chemotherapy can reduce the side effects of chemotherapy, but they are still drug resistant after a period of use [6–8]. The use of molecular diagnosis and treatment has become increasingly important to the treatment of non-small cell lung cancer in recent years [9, 10]. As a result, it is imperative to pinpoint the precise molecular mechanisms of the occurrence, development, invasion, and metastasis of NSCLC. In view of this, the development of new molecular biomarkers is vital for early diagnosis, prevention, and precision treatment.

It is increasingly common to use bioinformatics to identify biomarkers associated with certain diseases. At present, the bioinformatics technology provides a basis for further research on disease pathways and cellular activity networks by exploiting the underlying genetic and molecular mechanisms of disease. In this study, microarray datasets GSE118370 and GSE10072 were obtained from the Integrated Gene Expression Database (GEO) to identify differentially expressed genes (DEGs) between lung adenocarcinoma and adjacent normal tissues consisting of gene expression data for 66 lung adenocarcinoma samples of patients and 55 samples of normal lung tissue. In order to gain a deeper understanding of how these DEGs function biologically, pathway enrichment analysis using the Genomics Ontology (GO) and Kyoto Encyclopedia of Genes and Genomes (KEGG) was conducted. In addition, we created a protein-protein interaction (PPI) network related to DEGs.

With the help of String's online tool, the protein interaction between DEGs was constructed and then beautified with Cytoscape. Then, we searched for the hub gene using the CytoHubba plugin in Cytoscape. For this study, we used four different models to identify the hub genes that were most significant. In the last step, we will use tools such as UALCAN, *cBioPortal*, String, Cytoscape, and Timer to discover the gene and to learn about its role in the biological system. We demonstrated that SPP1 and PIK3R1 were possible biomarkers of lung adenocarcinoma. Further investigation revealed which we were pleasantly surprised to find that both SPP1 and PIK3R1 were associated with lung adenocarcinoma prognoses and immunotherapy. In conclusion, this study aims to develop promising new biomarkers from a new perspective for the diagnosis, prognosis, and molecular target therapy or immunotherapy of NSCLC.

## 2. Methods

### 2.1. Data Resources

**Figure 1:**
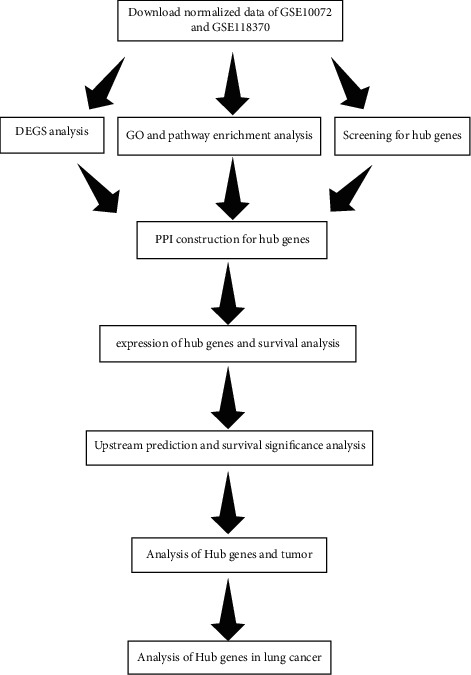
Flow chart of data preparation, processing, analysis, and validation.

The NCBI-GEO database (https://www.ncbi.nlm.nih.gov/geo/) [11] contains microarrays, chips, and gene expression data, which is an open high-throughput functional genome database. The microarray data of GSE10072 data (including 58 lung adenocarcinoma samples and 49 normal lung tissue samples) and GSE118370 data (including 6 lung adenocarcinoma samples and 6 normal lung tissue samples) are based on GPL96-57554 and GPL570-55999 platforms from the NCBI-GEO database (Figure 1).

The Limma package in R language was used to identify genes that differ in expression between lung cancer and normal lung tissues [12]. The DEGs were screened using the *R* studio's limma package, which performs log2 conversion and normalization of the matrix data. Through the Affy package, the GSE10072 and GSE118370 raw microarray data were processed in the *R* studio and standardized by the RMA method. The cutoff criteria were adjusted to *P* < 0.05, (logFC) which was >1 or <−1. DEGs were visualized using a hierarchical clustering heat map and a volcano map.

We used the GEPIA website (https://gepia.cancer-pku.cn) to probe the DEGs of lung adenocarcinoma in TCGA database [13]. In this research, DEGs were defined as *P* value <0.05, and the (logFC) value was >1 or <−1. Then, funrichVenn software was used to integrate the two data sets to obtain the common up-regulated and down-regulated DEGs.

### 2.2. GO and Pathway Enrichment Analysis

The pathway enrichment analysis was performed to determine the biological functions of the overlapping DEGs, based on FUNRICH software. We used the online WebGestalt (https://www.webgestalt.org/) tool for GO (Gene Ontology) enrichment, which is an extensively used method to investigate the molecular function (MF), cell component (CC), biological process (BP), and site of expression of genes or gene products for the analysis of DEGs [14]. The KEGG pathway was analyzed by the CLUGO [15] plugin in Cytoscape software (https://www.cytoscape.org/) [16]. PANTHER and REACTOME [17, 18] were also widely used databases for systematic analysis of high-level gene functions by KOBAS, using the CLUGO plugin in Cytoscape software. Significant pathways with *P* value <0.05 and the top 20 plotted were visualized by the R language.

### 2.3. PPI Network Establishment and Module Analysis

To better illustrate the existence of potential interactive relationships between overlapping DEGs, we selected the online database search tool String (https://string-db.org/) to retrieve interacting genes [19]. The first step is to draw the PPI network diagram for DEGs using the String website. Then, Cytoscape was applied to identify the top 30 genes based on the DEGREE, MCC, DMNC, and MNC methods generated by CytoHubba [20] plugin in Cytoscape software. The top common genes were identified according to the previously mentioned four methods which were selected as hub genes using FUNRICH software.

### 2.4. Expression and Survival Analysis of Hub Genes

To confirm differential expression of hub genes between lung adenocarcinoma and normal lung tissues, we validated each hub gene in TCGA database by using the online tool GEPIA website (https://gepia.cancer-pku.cn). Correlation between DEG expression and overall survival (OS) in lung adenocarcinoma was also analyzed using the same tool website on the GEPIA online database. *P* value <0.05 was considered statistically significant.

### 2.5. Upstream miRNA Prediction and Survival Analysis

Upstream miRNA prediction and survival analysis of the three genes (SPP1, PIK3R1, and PECAM1) were conducted with the miRtarbase, and the miRNA survival analysis was used by the OncoLnc (https://www.oncolnc.org/) [21] website. *P* value <0.05 was considered statistically significant.

### 2.6. Analysis of the Target Gene

Based on survival analysis, we analyzed the target gene which was identified from the hub genes using the UALCAN (https://ualcan.path.uab.edu/index.html) [22], GEPIA, STRING, and DAVID [23] online websites. In order to get the distribution and relationship of all proteins, we used the Human Protein Atlas (https://www.proteinatlas.org/) [24]. The protein expression level of target genes was verified in the Human Protein Atlas. *P* value <0.05 was considered statistically significant.

### 2.7. mRNA Expression Correlates with Immune Cell Infiltration and Immune Checkpoint Activation

The TIMER website (https://cistrome.shinyapps.io/timer/) [25] focuses on analyzing tumor immune relationships. TIMER was used to analyze mRNA expression data of SPP1 in TCGA database which was correlated with tumor infiltration and immune checkpoints. *P* value <0.05, and the (logFC) value was >1 or <−1 was considered statistically significant.

## 3. Results

### 3.1. Identification of DEGs in Lung Adenocarcinoma

**Figure 2:**
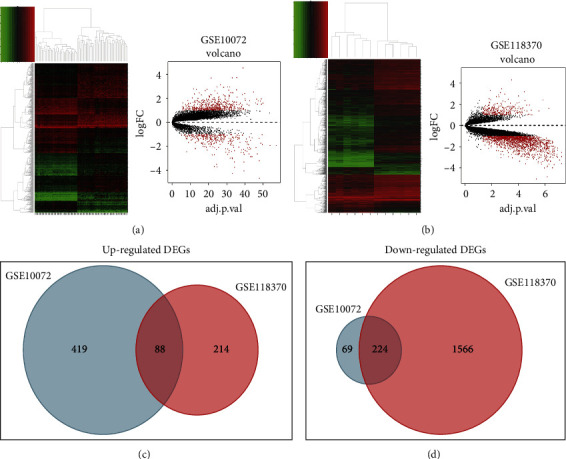
Identify DEGs shared between the two databases. (a) The heat map and volcano map of GSE10072. (b) The heat map and volcano map of GSE18370. (c) A Venn diagram used to identify 88 promising up-regulated target genes in lung adenocarcinoma. (d) A Venn diagram is used to identify 224 promising down-regulated target genes in lung adenocarcinoma.

In this study, we obtained gene expression profiles from lung carcinoma samples and normal lung tissues from the GSE10072 and GSE118370 datasets, as well as analyses of DEGs from TCGA database for lung carcinoma samples and normal lung tissues. A hierarchical clustering heat map and a volcano map were created to visualize DEGs (Figures 2(a) and 2(b)). By using *R* studio, we identified 800 and 2092 DEGs from GSE10072 and GSE118370 using the cutoff criterion of *P* = 0.05 and (logFC) > 1, respectively. Employing Venn analysis by FUNRICH software, we identified 88 up-regulated DEGs and 224 down-regulated DEGs both in GSE10072 and GSE118370 (Figures 2(c) and 2(d)).

### 3.2. Pathway Enrichment Analysis

**Figure 3:**
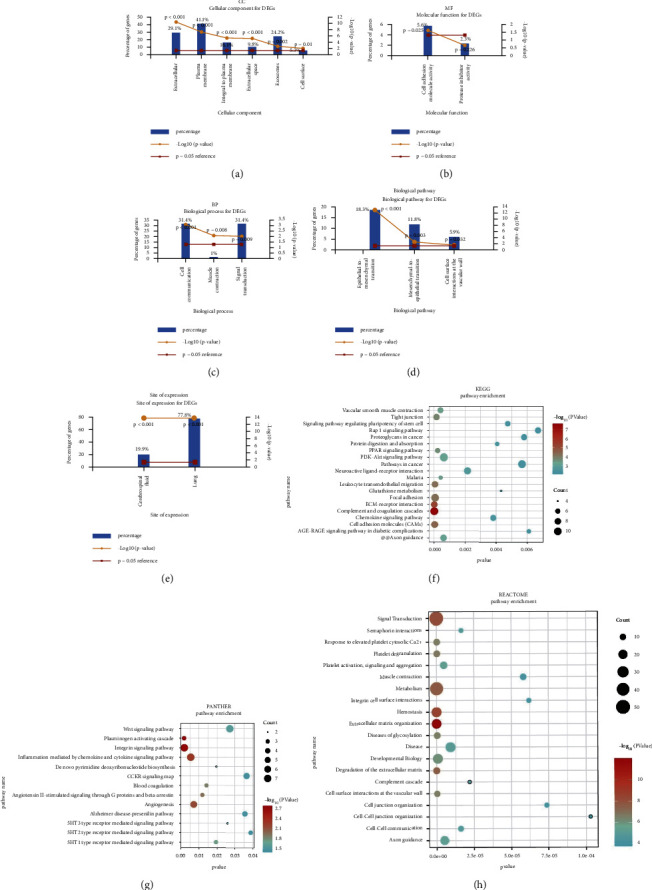
Pathway analysis of DEGs based on FUNRICH software. (a–e) GO analysis of CC, MF, BP, biological pathway, and site of expression. (f–h) Bubble diagram of KEGG, PANTHER, and the REACTOME pathway of lung adenocarcinoma. Significant pathways with *P* value <0.05 and top 20 were plotted by R language.

To initially identify the biological classification of DEGs, we used the GO analysis (three methods: CC, MF, and BP), biological pathway, site of expression pathway, KEGG, PANTHER, and REACTOM through FUNRICH software. A major change of DEGs in CC occurs at the extracellular level, plasma membrane level, integral plasma membrane level, extracellular layer, exosome level, and cell-surface level. The differentiation of DEGs in MF is mainly in cell adhesion activity, and there is no differentiation in protease inhibitor activity. The discrepancy of DEGs in the BP is extraordinarily enriched in cell communication, muscle contraction, and signal transduction (Figures 3(a)–3(c)). Based on the biological pathway, DEGs were enriched to epithelial-mesenchymal transitions as well as cell-surface interactions in the vessel wall (Figure 3(d)). While using the site of the expression pathway, the DEGs are focusing in cerebrospinal and lung (Figure 3(e)). The enrichment of the DEGs is mostly in complement and coagulation cascades, ECM-receptor interaction, cell adhesion molecules (CAMS), focal adhesion, and so on when using KEGG pathway analysis (Figure 3(f)). PANTHER analysis shows the DEGs are mostly enriched for signaling pathways related to integrin signaling, inflammation mediated by chemokine, chemokine signaling, plasminogen activating cascade, and so on (Figure 3(g)). At last, the DEGs are mostly enriched in extracellular matrix organization, signal transduction, metabolism, and so on when using REACTOME pathway analysis (Figure 3(h)).

### 3.3. PPI Construction and Screening for Hub Genes

**Figure 4:**
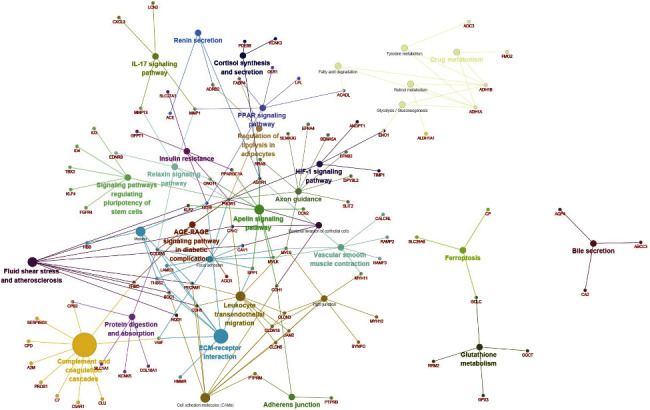
The results of KEGG and the REACTOME pathway analysis with the CLUGO plugin in Cytoscape software.

Our analysis was based on the STRING online tool and Cytoscape software; we constructed a DEG PPI network containing 94 DEGs with 94 nodes and 224 edges identified, as shown in Figure 4. Further analysis of these genes and pathways, as shown in the PPI network, CPB2, SERPING1, CFD, A2M, PROS1, C7, C5AR1, CLU, and THBD, is in the complement and coagulation cascades pathway. Some genes such as HMMR, VWF, SPP1, CD36, LAMC3, THBS2 SDC1, and so on are in the ECM-receptor interaction pathway, while some genes are the core status for many pathways across them. For example, PIK3R1 gene has 12 pathways, such as insulin resistance, relaxin signaling pathway, signaling pathway regulating stem cell pluripotency, fluid shear stress and atherosclerosis, AGE-RAGE signaling pathway in diabetic complication, focal adhesion, and so on.

**Figure 5:**
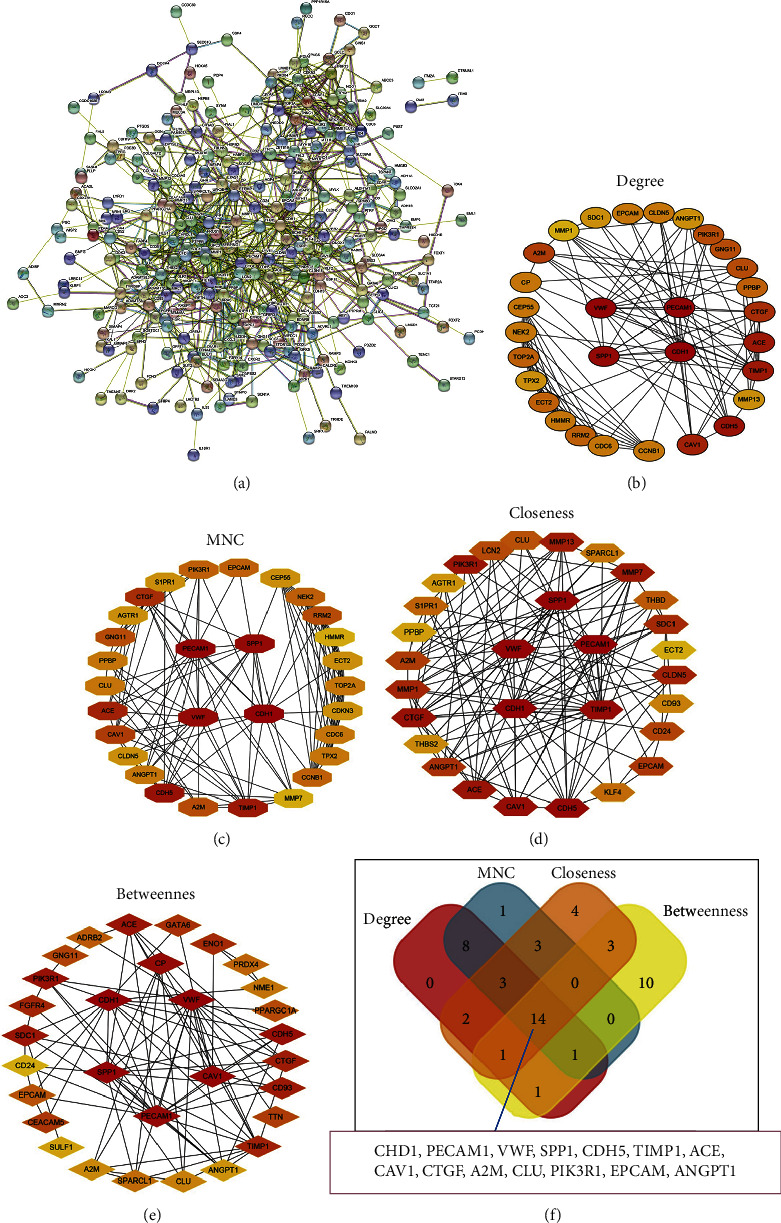
Determining the hub genes. (a) PPI network of 312 promising target genes in lung adenocarcinoma based on the string website. (b–e) Find hub genes with the CytoHubba plugin in Cytoscape software. Four different metrics were used: DEGREE, MNC, closeness, and betweenness. (f) A Venn diagram used to identify 14 hub genes in lung adenocarcinoma.

In order to study the protein networks associated with these genes, we created a DEG PPI network based on the STRING online database and Cytoscape (Figure 5(a)). The top 20 genes are identified as the most promising hub genes using CytoHubba plugin in Cytoscape software according to the degree, MNC, closeness, and betweenness methods, respectively (Figures 5(b)–5(e)). The genes are selected from the top most connected genes using Venn analysis, and 14 genes are obtained as hub genes which include CDH1, PECAM1, VWF, SPP1, CDH5, T1MP1, ACE, CAV1, CTGF, A2M, CLU, PIK3R1, EPCAM, and ANGPT1 (Figure 5(f)).

### 3.4. Expression of Hub Genes and Survival Analysis

**Figure 6:**
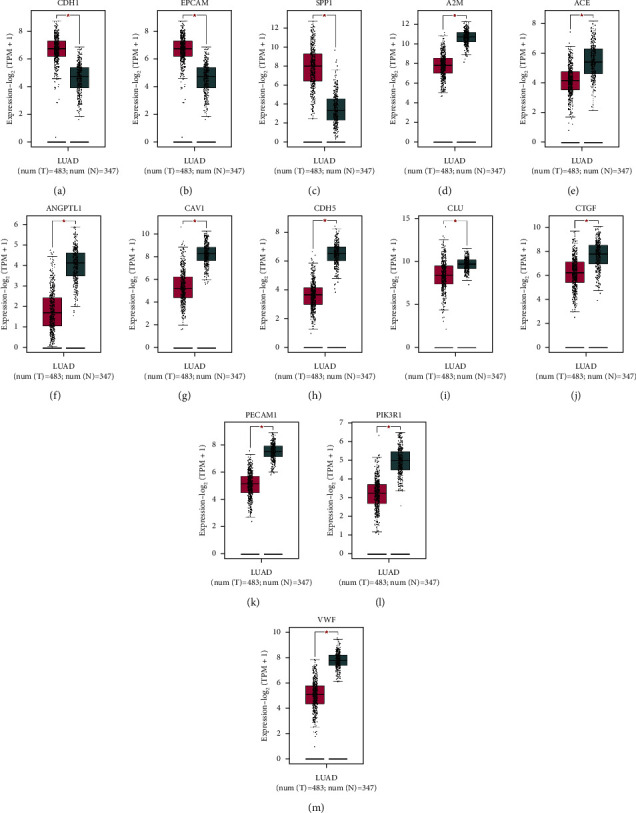
Expression analysis of 13 hub genes in lung adenocarcinoma based on GEPIA. (a) CDH1, (b) EPCAM, (c) SPP1, (d) A2M, (e) ACE, (f) ANGPTL1, (g) CAV1, (h) CDH5, (i) CLU, (j) CTGF, (k) PECAM1, (l) PIK3R1, and (m) VWF; *P* < 0.05 was considered as statistically significant.

To validate the distinctive expression of the 14 hub genes we got from previse analysis between lung adenocarcinoma (LUAD) and normal tissues. As a result of using the GEPIA website to analyze the expression level of each hub gene, we discovered that 13 hubs were significantly changed in LUAD (Figure 6). There is no statistical difference in gene TIMP1. The up-regulated genes are CDH1, EPCAM, and SPP1 (Figures 6(a)–6(c)), whereas the down-regulated genes are A2M, ACE, ANGPT1, CAV1, CDH5, CLU, CTGF, PECAM1, PIK3R1, and VWF (Figures 6(d)–6(m)).

**Figure 7:**
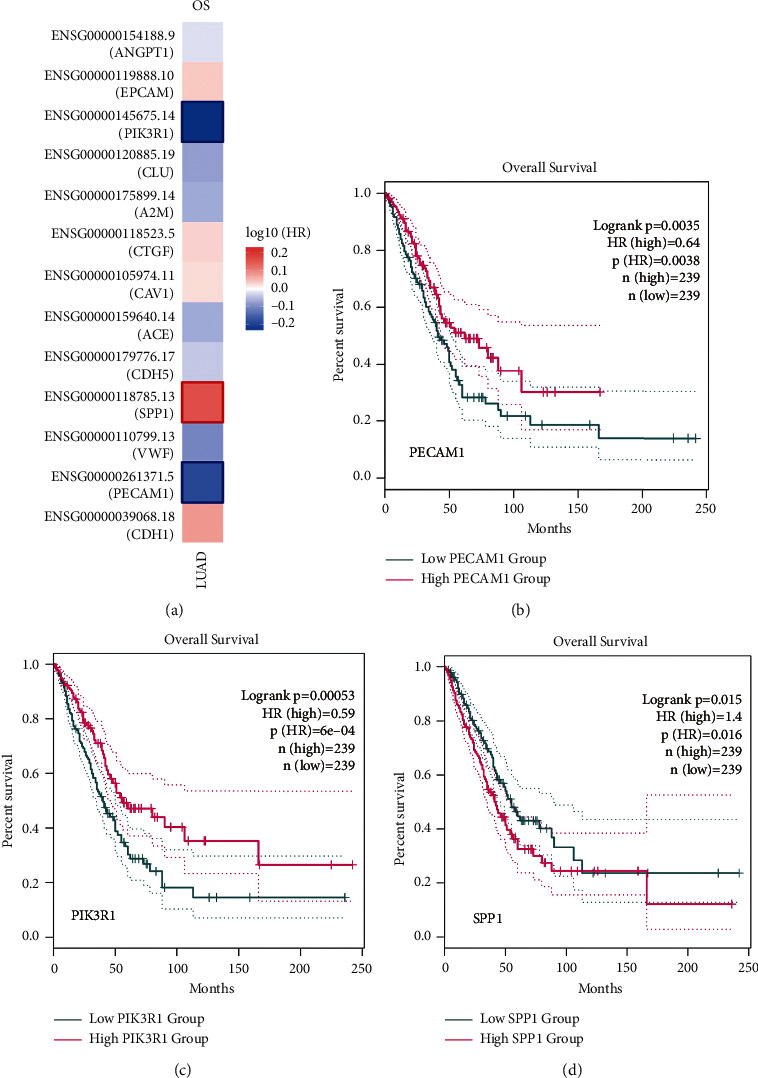
(a) Survival analysis of 14 hub genes in lung adenocarcinoma based on GEPIA. (b–d) We found that three genes (PECAM1, PIK3R1, and SPP1) were significantly correlated with overall survival. *P* < 0.05 was considered as statistically significant.

We carried out a survival analysis of the hub genes by the GEPIA website to investigate the relevance to the survival with lung adenocarcinoma patients. Three genes (PIK3R1, SPP1, and PECAM1) have a clear correlation with OS in lung adenocarcinoma patients (Figure 7(a)). Among the three genes, SPP1 is highly expressed in lung adenocarcinoma compared with normal lung tissue, but PECAM1 and PIK3R1 have low expression in lung adenocarcinoma compared with normal lung tissue, and the high expression of SPP1 is associated with poor prognosis (Figure 7(d)), while a positive prognosis is associated with high PECAM1 and PIK3R1 expression (Figures 7(b) and 7(c)).

### 3.5. Upstream microRNA Prediction and Survival Significance Analysis

**Figure 8:**
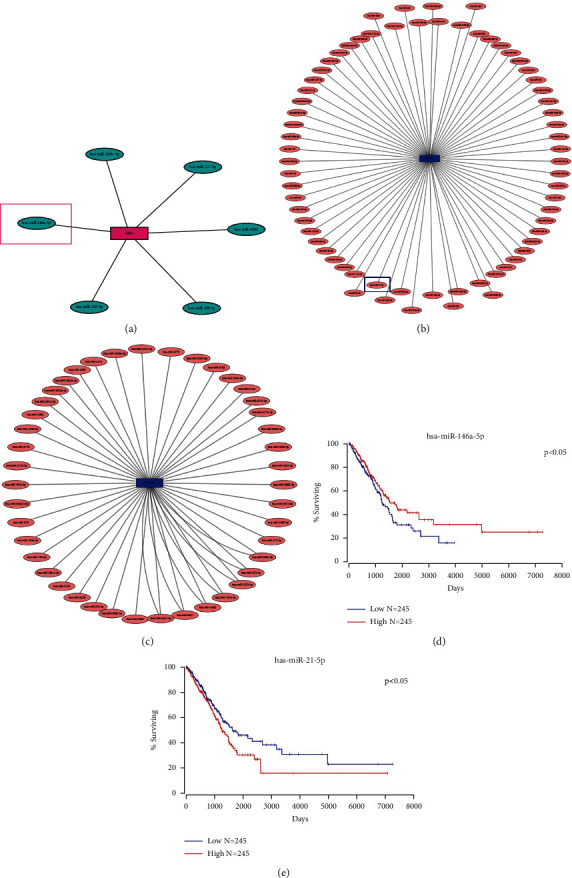
Upstream miRNA prediction and survival analysis of the three genes were conducted with the miRtarbase. (a) SPP1. (b) PIK3R1. (c) PECAM1. (d, e) miRNA survival analysis used the OncoLnc website, and hsa-miR-146a-5p and hsa-miR-21-5p were significantly correlated with overall survival adenocarcinoma.

The miRtarbase prediction tool was used to predict upstream miRNAs of three genes (SPP1, PIK3R1, and PECAM1) and found two miRNAs related to the two hub genes; miR-146a-5p is associated with SPP1, while miR-21-5p is associated with PIK3R1. However, we don't find any miRNAs significantly related to PECAM1 (Figures 8(a)–8(c)). Then, we analyzed the survival curves of miRNAs by the OncoLnc website. We found that the higher expression of miR-146a-5p on upstream of SPP1 is associated with a higher surviving rate in cancer patients (Figure 8(d)). On the contrary, lower miR-21-5p on upstream of PIK3R1 is associated with a higher surviving rate in cancer patients (Figure 8(e)), *P* < 0.05.

### 3.6. The Biological Role of PIK3R1 in Tumors

**Figure 9:**
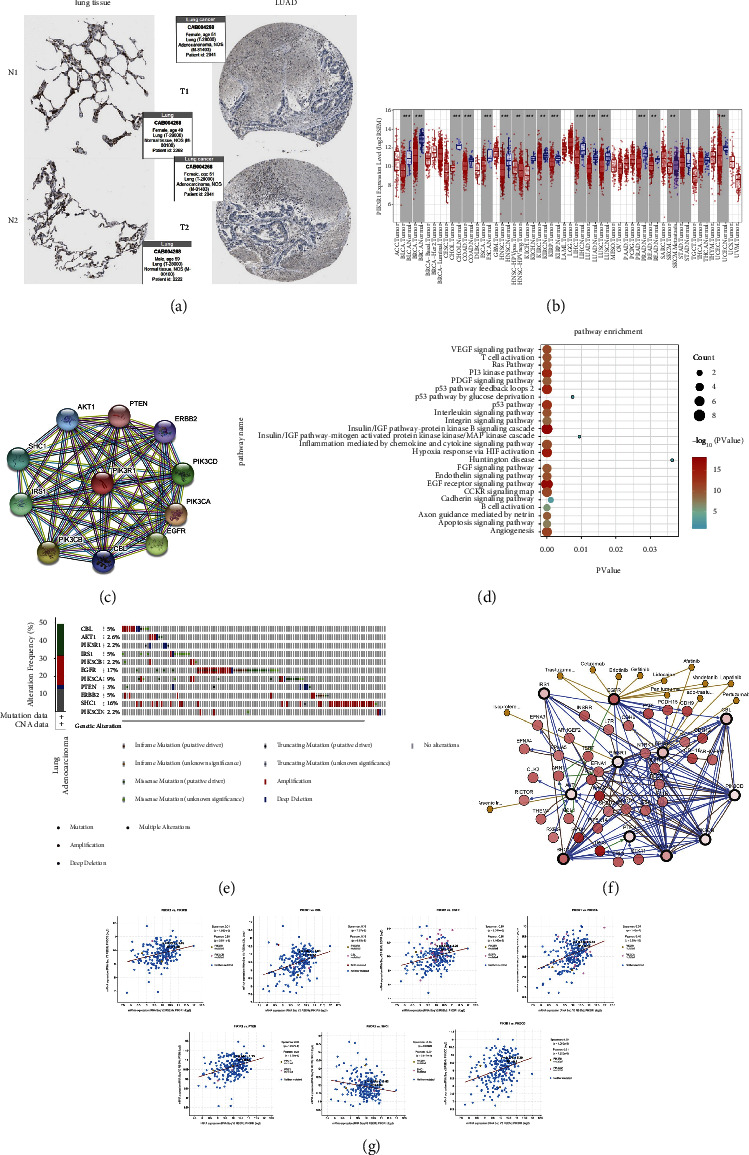
The biological role of PIK3R1 in tumors. (a) Immunohistochemical analysis of normal lung tissues and lung adenocarcinoma with the HPA online tool, and PIK3R1 was found to be expressed lower in LUAD tissues. (b) Expression of PIK3R1 in various tumors. (c) Interacting proteins for PIK3R1 gene STRING interaction network preview (showing top 10 STRING interactants). (d) Bubble diagram of the PANTHER pathway of PIK3R1 gene. Significant pathways with P value <0.05 and top 24 were plotted by R language. (e) Variation of PIK3R1 related genes in lung adenocarcinoma. (f) Analysis of the network of regulatory pathways of the 11 genes. White is for tumortargeted drugs, and yellow is for oncology drugs approved by the FDA. (g) The scatter plot showed the correlation between PIK3R1 expression and 7 hub gene signature.

PIK3R1 was identified as one of the target genes prior to survival analysis. Here, we analyzed PIK3R1 through the UALCAN website and found that PIK3R1 was highly expressed in lung adenocarcinoma compared with normal lung tissue (Figure 9(a)). The expression of PIK3R1 in different tumors and normal tissues by PAN cancer analysis on the TIMER website revealed that PIK3R1 was highly expressed in almost all the tumors (Figure 9(b)). 10 genes were found to be closely related to PIK3R1 by protein interaction analysis through the STRING website, AKT1, PTEN, ERBB2, PIK3CD, PIK3CA, EGFR, CBL, PIK3CB, IRS1, and SHC1 (Figure 9(c)). KEGG analysis of PIK3R1 showed that the pathway was mainly enriched in the EGF-receptor signaling pathway, insulin/IGF pathway-protein kinase B signaling cascade, p53 pathway, and p53 pathway feedback loops 2 (Figure 9(d)). The mutations and mutation rates of the 11 genes included PIK3R1 that were obtained by the *cBioPortal*  TCGA online analysis tool, and each gene is mutated (Figure 9(e)). Analysis of 10 gene-related pathway network was performed, with white representing tumor-targeted drugs and yellow representing oncology drugs approved by the FDA (Figure 9(f)). We next analyzed the correlation between PIK3R1 and seven genes associated with targeted drugs. We found that PIK3R1 has strong coexpression relationship with PIK3CB, CBL, EGFR, PIK3CA, PTEN, and PIK3CD, while it has negative relationship with SHC1 (Figure 9(g)).

### 3.7. The Biological Role of SPP1 in Tumors

**Figure 10:**
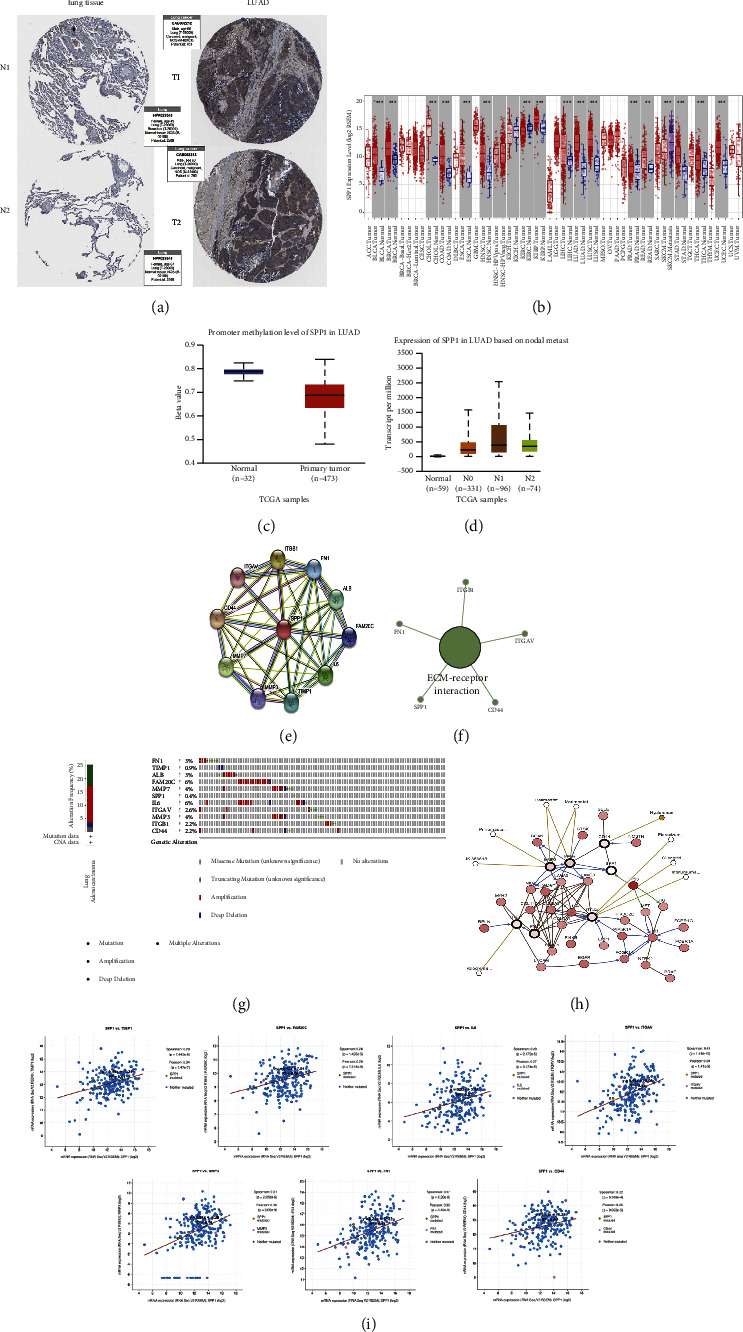
The biological role of SPP1 in tumors. (a) Immunohistochemical analysis of normal lung tissues and lung adenocarcinoma with the HPA online tool, and SPP1 was found to be highly expressed in LUAD tissues. (b) Expression of SPP1 in various tumors. (c) The methylation level of SPP1 in normal lung tissues and lung adenocarcinoma. (d) Expression of SPP1 in lung adenocarcinoma based on nodal metastasis status. (e) Interacting proteins for SPP1 gene STRING interaction network preview (showing top 10 STRING interactants). (f) Beautify the results of KEGG pathway analysis with the CLUGO plugin in Cytoscape software. (g) Variation of SPP1 related genes in the lungs. (h) Analysis of the network of regulatory pathways of the 11 genes. The white is for tumor-targeted drugs, and yellow is for oncology drugs approved by the FDA. (i) The scatter plot showed the correlation between the SPP1 expression and the 7 hub gene signature.  ^*∗*^*P* < 0.05,  ^*∗∗*^*P* < 0.01, and  ^*∗∗∗*^*P* < 0.001.

The difference expression of SPP1 in lung adenocarcinoma and normal tissues was analyzed by the HPA online tool. Surprisingly, SPP1 was highly expressed in lung adenocarcinoma compared to the normal lung tissue (Figure 10(a)). The expression of SPP1 in different tumors and normal tissues by PAN cancer analysis on the TIMER website revealed that SPP1 is highly expressed in almost all tumors (Figure 10(b)). The methylation level of SPP1 in lung adenocarcinoma is significantly decreased (Figure 10(c)) which is determined by the UALCAN online tool. SPP1 is also associated with clinical lymph node metastasis. The SPP1 is most expressed in grade *N*3 lymph node metastasis compared to *N*0, *N*1, and *N*2 (Figure 10(d)). 10 genes closely related to SPP1 are found by SPP1 protein interaction analysis through the STRING website (Figure 10(e)). KEGG analysis of SPP1 revealed that 10 genes were closely related to it and these genes were mainly enriched in the ECM-receptor interaction pathway (Figure 10(f)). The mutations and mutation rates of these 11 genes that included SPP1 were obtained by the *cBioPortal*  TCGA online analysis tool, and each gene is mutated (Figure 10(g)). Analysis of 11 gene-related pathways network was performed, with white representing tumor-targeted drugs and yellow representing oncology drugs approved by the FDA (Figure 10(h)). The relationship between these genes and SPP1 was also analyzed. We found that SPP1 has strong coexpression relationship with TIMP1, FAM20C, IL6, ITGAV, MMP3, FN1, and CD44 (Figure 10(i)).

### 3.8. SPP1 Acts as Immune-Related Genes in Lung Adenocarcinoma

**Figure 11:**
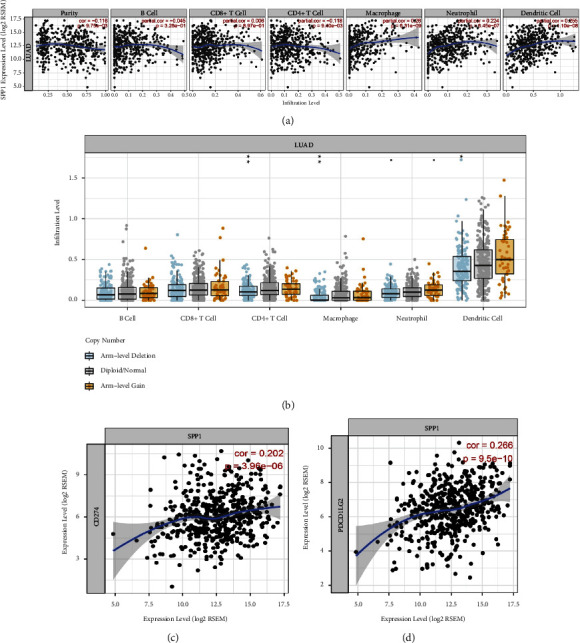
Immune correlation analysis in lung adenocarcinoma based on the TIMER website. (a) Relationship between SPP1 and immune cells. (b) The expression of immune cells in lung adenocarcinoma (LUAD). (c) Relationship between SPP1 and CD274 checkpoints. (d) Relationship between SPP1 and PDCD1LG2 checkpoints.

In order to explore the relationship between lung adenocarcinoma and tumor immunity, we analyzed the immune cell infiltration and found that SPP1 is expressed in many immune cells, such as macrophages, and the association of neutrophils and dendritic cells in lung adenocarcinoma with SPP1 was analyzed using the GEPIA website (Figure 11(a)*P* < 0.05), while SPP1 was involved in the infiltration of CD4^+^ T cells, macrophage and dendritic cells through the TIMER website (Figure 11(b), *P* < 0.05). As immunotherapy is currently mainly focused on immune checkpoint inhibitors, such as CD274, PDCD1, PDCD1LG2, and CTLA4, we further analyzed the coexpression relationship of SPP1 and immune checkpoint-related genes. We are surprised to find that SPP1 has significant coexpression relationship with CD274 and PDCD1LG2 (Figures 11(c) and 11(d)).

## 4. Discussion

Lung cancer is one of the most common malignant tumors in the world, so it poses a serious threat to human health. Worldwide, lung cancer accounts for about 17% of all new cases of malignant tumors and about 23% of all patients with tumor-related death [26]. Its incidence and mortality are increasing year by year, ranking the first among malignant tumors [27, 28]. Finding out the molecular mechanism and biomarkers related to the occurrence and development of lung cancer has always been the focus and difficulty of clinical and scientific research, which has important research value in improving the diagnosis, treatment efficacy, and prognosis survival of lung cancer patients.

The aim of the present study is through the bioinformatics analysis which is performed systematically to clarify the key effect of the candidate genes and pathways in NSCLC. Gene expression data (expression profiles GSE118370 and GSE10072) were gained from the Gene Expression Omnibus database.

Subsequently, 1,635 and 633 potential DEGs were obtained and identified 88 up-regulated genes and 234 down-regulated genes overlapped in the two GSE datasets in lung adenocarcinoma. Then, we performed enrichment analyses of GO analysis (three methods: CC, MF, and BP), biological pathway, site of expression pathway, KEGG, PANTHER, and REACTOM to analyze up-regulated and down-regulated genes.

The results indicated that the main changes of DEGs in CC are mainly in the extracellular part, and the main differentiation of DEGs in MF is cell adhesion activity, and the main discrepancy of DEGs in the BP is being extraordinarily enriched in cell communication. The epithelial-to-mesenchymal transition had the highest enrichment score in the biological pathway, while the cerebrospinal parts and lungs had the highest enrichment score in the site of the expression pathway. The complement and coagulation cascades had the highest enrichment score in KEGG pathway analysis, and the integrin signaling pathway had the highest enrichment score in the PANTHER pathway analysis, and the extracellular matrix organization had the highest enrichment score in REACTOME pathway analysis.

Furthermore, we constructed a PPI network to analyze the interactional relationships between the DGEs which included 94 nodes and 224 edges. 14 hub genes were identified with three up-regulated genes (CDH1, EPCAM, and SPP1) and ten down-regulated genes (A2M, ACE, ANGPT1, CAV1, CDH5, CLU, CTGF, PECAM1, PIK3R1, and VWF), and one gene had no significance. Surprisingly, the survival analysis revealed that three genes (PIK3R1, SPP1, and PECAM1) have a clear correlation with OS in lung adenocarcinoma patients. Among these three genes, SPP1 is highly expressed in lung adenocarcinoma compared with normal lung tissue, and the high expression is associated with poor prognosis; however, the poor prognosis was associated with the low expression of PECAM1 and PIK3R1. Therefore, we chose these three genes (PIK3R1, SPP1, and PECAM1) as target genes for the next analysis. These three genes have a clear correlation with OS in lung adenocarcinoma patients. SPP1 is highly expressed in lung adenocarcinoma compared with normal lung tissue, and the high expression of SPP1 is associated with poor prognosis, while the high expression of PECAM1 and PIK3R1 is associated with good prognosis. The results indicated that SPP1 is a cancer promoter (oncogene), while PECAM1 and PIK3R1 are cancer suppressor genes.

The oncoming analysis was performed with the survival curves of upstream miRNAs with three genes (PIK3R1, SPP1, and PECAM1), respectively. The results showed that the higher expression of miR-146a-5p on upstream of SPP1 is associated with a higher surviving rate in cancer patients (Figure 8(d)). On the contrary, lower miR-21-5p on upstream of PIK3R1 is associated with a higher surviving rate in cancer patients (Figure 8(e)), *P* < 0.05.

Platelet adhesion molecule-1 (PECAM1), a cell adhesion and signaling receptor, is located on chromosome 17q23.3 and encodes a protein found on the surface of monocytes, platelets, neutrophils, and certain types of T cells and makes up most of the intercellular junctions in endothelial cells. PECAM1 is a member of the immunoglobulin superfamily and is expressed in monocytes, neutrophils, macrophages, and other types of immune cells, as well as endothelial cells [29]. They may be involved in leukocyte migration, angiogenesis, and integrin activation. It was previously reported that PECAM-1 is also associated with advanced metastatic tumor progression [30]. Anti-PECAM-1 antibodies have been reported to inhibit late metastatic progression of various tumors without blocking tumor-platelet and tumor-endothelial interactions, events associated with the initial establishment of metastatic tumor foci. Studies of the molecular mechanism of PECAM-1 have shown that PECAM-1 mediates the release of soluble mediators that stimulate in vitro tumor cell proliferation [31]. Yu et al. determined that PECAM-1 plays a key role in tumorigenesis of LUAD by regulating vascular endothelial growth factor (VEGF) expression [32]. In this study, based on the survival and the hub gene analysis, we found that PECAM-1 expression was low in lung adenocarcinoma tissues, and importantly, PECAM-1 expression was associated with worse prognosis in lung adenocarcinoma. It may be an important predictor of prognosis.

Secretory phosphoprotein 1(SPP1) is a secretory acidic glycoprotein with multiple functions, also known as osteopontin (OPN). It contains the plant homeodomain (PHD) finger, which is a reading domain that typically binds unmethylated H3K4(H3K4me0), dimethylated H3K4(H3K4me2), or trimethylated H3K4(H3K4me3) [33]. It has been reported that it is a key extracellular matrix protein involved in tumor progression and metastasis and is considered as a promising biomarker for prognosis and therapeutic targets [34, 35]. However, it played different functions when in different cell locations. Patients with higher cytoplasmic SPP1 expression levels had a significantly better prognosis than those with lower SPP1 levels. However, when expressed in the nucleus, SPP1 did not show prognostic value in colorectal cancer [36].

This implies that SPP1 overexpression in the cytoplasm is an important and good prognostic biomarker. SPP1 enhances the drug resistance of the second generation EGFR TKI in lung cancer treatment. Inhibition of SPP1 may be a therapeutic target to overcome afatinib resistance [37]. At the same time, studies have found that SPP1 can regulate the expression of PD-L1 to mediate the immune escape of lung adenocarcinoma cells. In addition, SPP1 is also considered a marker of early lymphatic metastasis in lung cancer [38, 39]. This indicates that SPP1 plays an extremely important role in the progression of lung cancer and also provides strong supporting evidence for our findings.

Silencing SPP1 can inhibit the proliferation, invasion, migration, and the EMT process of gastric cancer cells by inhibiting the PI3K/AKT signaling pathway and promote the apoptosis of gastric cancer cells [40]. SPP1 mediates chemotherapy resistance in prostate cancer [41]. In addition, some scholars have demonstrated that inhibition of SPP1 expression can inhibit the progression of melanoma [42]. These evidences suggest that SPP1 plays a role in promoting the progression of various cancers. Therefore, we analyzed the expression of SPP1 in lung cancer and different cancers.

To investigate the relationship between SPP1 and lung adenocarcinoma, we performed immunohistochemistry by using the HPA online tool. It was found that SPP1 was highly expressed in lung adenocarcinoma compared with normal lung tissue. The results of the TIMER website analysis showed that SPP1 was highly expressed in almost all cancers. The methylation level of SPP1 is significantly decreased in lung adenocarcinoma, and SPP1 is clearly associated with clinical lymph node metastasis. We were surprised to find that the expression of SPP1 was associated with miR-146a-5p and the high expression of miR-146a-5p was related to good prognosis.

MicroRNAs (miRNAs) were named as a rank of small noncoding RNA molecules which are less than 22 nucleotides and induce post-transcriptional mRNA degradation after binding to the 3-untranslated regions (3′UTRs) in target mRNAs [43]. The expression level and regulatory mechanism of miR-146a-5p was diversity and have been reported in a variety of tumors. miR-146a-5p is lowly expressed in lung cancer, gastric cancer, and breast cancer [44–46], and the high expression of exogenous miR-146a-5p can inhibit the proliferation of tumor cells. However, this is in contrast to the high expression level of miR-146a-5p in melanoma and thyroid carcinoma [47, 48]. MiR-146a-5p was a powerful inhibitor in cervical cancer and epithelial ovarian cancer with a better prognosis for patients [49, 50]. This is consistent with our finding that Mir-146A-5p expression is indeed associated with lung adenocarcinoma. In addition, miR-146a-5p has good clinical application value, and the serum level of miR-146a-5p in patients with non-small cell lung cancer is lower, and the expression level indicates a poor prognosis [51]. MiR-146a-5p can also be used as a diagnostic indicator of non-small cell lung cancer [52].

PIK3R1 gene is the regulatory subunit coding gene of PI3K. An increasing number of PIK3R1 has been identified as differentially expressed in many human cancers and associated with tumor progression and metastasis [53]. PIK3R1 is abnormally expressed in a variety of tumors and is associated with increased cell proliferation and invasion. The mutation of PIK3R1 gene in breast cancer, endometrial cancer, and urothelial carcinoma can lead to pathogenesis [54–56]. However, in different types of cancers, it functions differently. In ovarian cancer and colon cancer, PIK3R1 gene played a role of an oncogene [57], while in hepatocellular carcinoma and breast cancer, it played as a tumor suppressor gene [58, 59]. When down-regulated expression of PIK3R1 gene can inhibit PTEN function and reduce the degradation of PIP3 molecule, which was activated PI3K/AKT signaling to play the role of the tumor suppressor gene [60]. The change of the PI3K/AKT signaling pathway is also related to the occurrence of breast cancer [61]. Studies have found that Mir-21 targeting PIK3R1 can inhibit the migration and invasion of tumor cells by reducing the PI3K/AKT signaling pathway and reversing EMT in breast cancer patients [62]. Although we identified the role of PIK3R1 in exacerbating NSCLC progression, the underlying mechanisms driving tumor progression need to be further elaborated. We therefore analyzed the biological processes in which it might participate. We also found in lung adenocarcinoma that the overexpression of PIK3R1 was associated with miR-21-5p and the low expression of miR-21-5p means good prognosis.

MiR-21-5p is the one unstable strand chain form from mature miR-21 while the other miR-21-3p chain is degraded. MiR-21 was considered an onco-miRNA which participated in oncogenesis via the regulation of a lot of tumor suppressors [63]. The study found that the expression level of Mir-21 in colorectal cancer tissues is higher than that in adjacent tissues, and miR-21-5p promoted proliferation and invasion in colon adenocarcinoma cells [64, 65]. MiR-21-5p induces cell proliferation by targeting TGFBI in non-small cell lung cancer cells [66]. It can inhibit the sensitivity of hepatocellular carcinoma cells to cisplatin [67]. Mir-21-5p has also been reported to be involved in a variety of signaling pathways. It promotes the progression of lung adenocarcinoma by targeting the SET/TAF-I*α* fraction [68]. It also reported MiR-21-5p promoted the occurrence of lung cancer by inhibiting the RAS/MEK/ERK pathway and inhibiting apoptosis [69]. Downregulation of miR-21-5p inhibited both proliferation and apoptosis in oesophageal squamous cell carcinoma cells via the CADM2/Akt pathway [70]. It promotes EGF-induced pancreatic cancer cell proliferation by targeting Spry2, and the mechanistic revealed that miR-21 targeted MAPK/ERK and PI3K/AKT signaling pathways to modulate cell proliferation [71]. In our study, we found that PIK3R1 was highly correlated with miR-21-5p and affected the prognosis of patients. Therefore, we believe that PIK3R1 may be regulated by miR-21-5p and participate in the malignant progression of non-small-cell lung cancer.

In our study, we found that SPP1 acted as an immune-related gene. We used the TIMER website to analyze the involvement of the infiltration of macrophages, neutrophils, and dendritic cells. The expression of immune cells was involved in the infiltration of CD4^+^ T cells, macrophages, and dendritic cells in lung adenocarcinoma (LUAD). Then, we further analyzed the coexpression of the relationship between SPP1 and immune checkpoint related genes. It was found that SPP1 has significant coexpression relationship with CD274(PD-L1) and PDCD1LG2(PD-1). The immune treatment with (PD-1/PD-L1) is a hot topic in recent years, and the therapeutic effect is encouraging. Studies have shown that PD-L1 was highly expressed in ovarian cancer, melanoma, non-small-cell lung cancer, renal cell cancer, and other tumors, and the expression of PD-1 in CD8^+^T cells infiltrated in tumor sites is higher than that in peripheral blood [72–75]. The combination of PD-L1 and PD-1 inhibited glycolysis, amino acid metabolism, and mitochondrial metabolism. In addition, it can promote the accumulation of polyunsaturated fat A(PUFA) and the oxidation and activation of fatty acids and finally change the metabolic mode of T cells [76, 77].

PD-1 was named the programmed death receptor because of its participation in apoptosis [78]. It is mainly expressed on the surface of T cells, B cells, and natural killer cells. Pd-1 consists of 288 amino acid residues and belongs to the CD28 family, which is located on the PDCD1 gene. Pd-l1, also known as CD274, is the major ligand of PD-1 (the other ligand is PD-L2), a 290-amino acid residue transmembrane protein that is mainly expressed in mature immune cells, such as CD4^+^T cells, CD8^+^T cells, B cells, macrophages, dendritic cells, endothelial cells, and other nonimmune cells [79]. Theses suggested that the PD-1/PD-L1 pathway was abnormally activated in tumors. Studies have shown that the PD-L1 expression in lung adenocarcinoma is associated with many genes and activates multiple pathways. KRAS up-regulated PD-L1 through p-REK instead of the p-AKT pathway, and the PD-1 blocker or ERK inhibitor can rescue the antitumor function of T cells and reduce the survival rate of KRAS mutated non-small-cell lung cancer cells [80]. The expression of PD-L1 can be down-regulated by the EGFR inhibitor erlotinib in lung cancer with EGFR receptor mutation [81]. The patients with positive PD-L1 were also more sensitive to EGFR tyrosine kinase inhibitors [82]. Studies have shown that chemotherapy, radiotherapy, the cytotoxic T-lymphocyte associated antigen 4 blocker (CTLA4), and other therapeutic methods which produce infiltrating T cells can up-regulate PD-L1 expression through IFNy produced by T cells, which is related to the STAT pathway [83–85].

## 5. Conclusions

This study aims to develop promising novel biomarkers from a new perspective for NSCLC diagnosis, prognosis, and molecular target therapy or immunotherapy. We reveal two potential biomarkers for lung adenocarcinoma, including PIK3R1 and SPP1. Apart from two microRNA, miR-21-5p and miR-146a-5p are related to the prognosis of lung adenocarcinoma which are consistent to PIK3R1 and SPP1. All of these genes take part in the regulation of biological activities in lung adenocarcinoma, providing a basis for improving detection and immunotherapeutic targets for lung adenocarcinoma. Finally, we found that the immune checkpoint-related genes CD274(PD-L1) and PDCD1LG2(PD-1) was related to SPP1 in lung adenocarcinoma. With the development of tumor immunology, tumor immunotherapy is expected to become the successor to surgery, chemotherapy, radiotherapy, and targeted therapy. The previous results also lay a very solid foundation for our nature research and clinical application. However, this study has some limitations, lack of in vivo and in vitro experimental verification. In the future work, we will be trying to study the further mechanisms in the base of bioinformatics.

## Data Availability

All data generated or analyzed during this study are included within this article.

## Supplementary Materials

Supplementary Materials Since the picture contains a lot of content. It may be difficult to see clearly because the legend is too small in the reading process. So, we provide the original data of the picture separately.
