# Supplementary material for: Identification of Key Biomarkers and Candidate Molecules in Non-Small-Cell Lung Cancer by Integrated Bioinformatics Analysis
Source: Genet Res (Camb). 2023 Jan 3;2023:6782732. doi: 10.1155/2023/6782732 (PMC9831708; doi:10.1155/2023/6782732)
Supplement: Supplementary Materials — Since the picture contains a lot of content. It may be difficult to see clearly because the legend is too small in the reading process. So, we provide the original data of the picture separately. [file 6782732.f1.doc]

Fig1.Identify DEGs shared between the two databases. (A) The heat map and volcano map of GSE10072. (B)The heat map and volcano map of GSE18370.(C)A Venn diagram used to identify 88 promising up-regulated target genes in lung adenocarcinoma. (D)A Venn diagram used to identify 224 promising down-regulated target genes in lung adenocarcinoma.

Fig2. Pathway analysis of DEGs based on FUNRICH software. (A, B, C, D, E) GO analysis of CC, MF,BP,Biological pathway and site of expression.(F,G,H)Bubble diagram of KEGG,PANTHER and REACTOME pathway of lung adenocarcinoma. Significant pathways with P-value < 0.05 and top 20 were plotted by R language.

Fig 3 The results of KEGG and REACTOME pathway analysis with the Clugo plugin in

Cytoscape software.

Fig4.Determining the hub genes. (A)PPI network of 312 promising target genes in lung adenocarcinoma based on string website. (B, C, D, E) Find hub gene with the Cytohubba plugin in

Cytoscape software. Four different metrics were used: DEGREE, MNC, Closeness and Bctweenness. (F) A Venn diagram used to identify 14 hub genes in lung adenocarcinoma.

Fig5.Expression analysis of 13 hub genes in lung adenocarcinoma based on GEPIA. (A) CDH1, (B) EPCAM, (C) SPP1, (D) A2M, (E) ACE, (F)ANGPTL1, (G) CAV1, (H)CDH5, (I)CLU, (J) CTGF, (K) PECAM1, (L) PIK3R1, (M) VWF; p <0.05 was considered as statistically significant.

Fig6.(A) Survival analysis of 14 hub genes in lung adenocarcinoma based on GEPIA.(B,C,D) We found that three genes(PECAM1,PIK3R1,SPP1) were significantly correlated with overall survival. p <0.05 was considered as statistically significant.

Fig7.Upstream miRNA prediction and survival analysis of the three genes were conducted with the miRtarbase.(A)SPP1.(B)PIK3R1.(C)PECAM1.(D,E) miRNA survival analysis used oncolnc website, hsa-miR-146a-5p and hsa-miR-21-5p were significantly correlated with overall survival .

adenocarcinoma. (H) Analysis of the network of regulatory pathways of the 11 genes. the white is for tumor-targeted drugs and yellow is for oncology drugs approved by the FDA. (I)Scatter plot showed the correlation between the SPP1 expression and the 7 hub genes signature. *P<0.05, **P<0.01, ***P<0.001.

Fig8.The biological role of PIK3R1 in tumors. (A)Immunohistochemical analysis of normal lung tissues and lung adenocarcinoma with HPA online tool, and PIK3R1 was found to be lower expressed in LUAD tissues.(B)Expression ofPIK3R1 in various tumors. (C)Interacting Proteins for PIK3R1 Gene STRING Interaction Network Preview (showing top 10 STRING interactants).(D) Bubble diagram of PANTHER pathway of PIK3R1gene . Significant pathways with P-value < 0.05 and top 24 were plotted by R language. (E) Variation of PIK3R1 related genes in lung adenocarcinoma. (H) Analysis of the network of regulatory pathways of the 11 genes. the white is for tumor-targeted drugs and yellow is for oncology drugs approved by the FDA. (I)Scatter plot showed the correlation between the PIK3R1 expression and the 7 hub genes signature.

Fig9.The biological role of SPP1 in tumors. (A)Immunohistochemical analysis of normal lung tissues and lung adenocarcinoma with HPA online tool, and SPP1 was found to be highly expressed in LUAD tissues.(B)Expression of SPP1 in various tumors. (C) methylation level of SPP1 in normal lung tissues and lung adenocarcinoma.(D) Expression of SPP1 in lung adenocarcinoma based on nodal metastasis status.(E)Interacting Proteins for SPP1 Gene STRING Interaction Network Preview (showing top 10 STRING interactants).(F) Beautify the results of KEGG pathway analysis with the Clugo plugin in Cytoscape software. (G) Variation of SPP1 related genes in lung.

Fig10 immune correlation analysis in lung adenocarcinoma based on the TIMER website. (A) Relationship between SPP1 and immune cells. (B) The expression of immune cells in lung adenocarcinoma(LUAD). (C) Relationship between SPP1 and CD274 checkpoints. (D) Relationship between SPP1 and PDCD1LG2 checkpoints.
